# Pharmacologic or genetic activation of SIRT1 attenuates the fat-induced decrease in beta-cell function in vivo

**DOI:** 10.1038/s41387-019-0075-z

**Published:** 2019-03-19

**Authors:** Tejas Desai, Khajag Koulajian, Aleksandar Ivovic, Danna M. Breen, Lemieux Luu, Evangelia L. Tsiani, Michael B. Wheeler, Adria Giacca

**Affiliations:** 1 Department of Physiology, University of Toronto, Toronto, ON Canada; 2 Faculty of Applied Health Sciences, Brock University, St. Catherines, ON Canada; 3 Department of Medicine, University of Toronto, Toronto, ON Canada; 4 Banting and Best Diabetes Centre, University of Toronto, Toronto, ON Canada; 5 Institute of Medical Sciences, University of Toronto, Toronto, ON Canada

## Abstract

**Background:**

There is evidence that sirtuin 1 (SIRT1), a key regulator of nutrient metabolism, increases β-cell secretory function. Excess circulating fat, as seen in obesity, has been shown to decrease β-cell function, an effect that may involve decreased SIRT1 activity. Consequently, SIRT1 activation may increase β-cell function in conditions of elevated plasma-free fatty acid levels. Here we attempted to attenuate the lipid-induced decrease in β-cell function in vivo using pharmacological and genetic models of SIRT1 activation.

**Methods:**

Our pharmacologic model involved 48 h intravenous infusion of Wistar rats with either saline or oleate with or without the SIRT1 activator resveratrol. Additionally, we used β-cell-specific SIRT1 overexpressing (BESTO) mice and wild-type littermates infused for 48 h intravenously with either saline or oleate. In both models, the infusion period was followed by assessment of β-cell function using the hyperglycemic clamp method.

**Results:**

Lipid infusion resulted in a significant decrease in β-cell function as expected in both rats (*p* < 0.05) and mice (*p* < 0.001). Both models of SIRT1 activation, which did not alter β-cell function in the absence of fat, resulted in partial protection from the fat-induced decrease in β-cell function (NS vs. control).

**Conclusion:**

These results suggest that SIRT1 is a therapeutic target in decreased β-cell function specifically induced by fat.

## Introduction

Over the last decade it has become quite evident that sirtuins and SIRT1 are key factors in the regulation of nutrient metabolism. At the same time, our lab and others have demonstrated that prolonged exposure to lipids plays a crucial role in decreasing β-cell function. However, there has been limited research connecting these two areas of investigation.

A few key studies have examined the importance of SIRT1 within the β-cell and demonstrated that SIRT1 overexpression or knock-out mouse models display increased or decreased insulin secretion respectively^1,2^. These studies attributed the changes in insulin secretion to changes in UCP2, which is a transcriptional target that has been shown to be repressed by SIRT1. Subsequent studies further demonstrated that a lack of NAD availability, which is essential to sirtuin function, results in decreased SIRT1 activity and decreased β-cell function^3^. More recently, β-cell-specific SIRT1 knockout models exhibited decreased glucose-stimulated insulin secretion (GSIS), but interestingly no changes in UCP2 expression^4,5^.

We have shown that oxidative stress is a mediator of fat-induced decrease in β-cell function in vitro and in vivo^6–9^, and a couple of studies linked this notion to SIRT1 by demonstrating that oxidative stress-induced DNA damage could activate NAD-dependent PARP enzymes, thus reducing NAD availability for SIRT1 and ultimately SIRT1 activity^10,11^. Changes in SIRT1 activity result in changes in SIRT1 expression by a feed forward mechanism^12^, and in vitro studies have shown decreased SIRT1 expression in islets after exposure to palmitate and prevention of palmitate-induced decrease in insulin secretion using the SIRT1 activator resveratrol^13^. Currently, whether SIRT1 activation can prevent the fat-induced decrease in β-cell function in vivo is not known. However, based on the relationship between sirtuins and oxidative stress^14^ and the evidence from the above-described in vitro data^13^, it can be hypothesized that either pharmacological or genetic activation of SIRT1 could prevent fat-induced decrease in β-cell function in vivo.

## Materials and methods

### Animals

All procedures were in accordance with the Canadian Council of Animal Care Standards and were approved by the Animal Care Committee at the University of Toronto. Female Wistar rats (250–300 g, purchased from Charles River, QC, Canada) and male beta-cell specific Sirt1 overexpressing (BESTO) transgenic mice (TG) (25–35 g, obtained from Dr. S-I. Imai, St. Louis, MO, USA) or wild type (WT) littermates were used in all studies. All animals were housed in the University of Toronto’s Department of Comparative Medicine. They were exposed to a 12 h light/dark cycle and were fed a Teklad Global diet (#2918, Harland Teklad Global Diets, Madison, WI, USA).

### Studies in rats

#### Surgery

A jugular vein and the contralateral carotid artery of the rats were cannulated as previously described^6^.

#### Intravenous infusions

After 3–4 days post-surgery recovery the rats were randomly assigned to 48 h infusion with either: (1) saline (SAL; 154 mmol/l) as control, (2) oleate (OLE; 1.4 μmol/min, Sigma, St. Louis, MO, USA) to elevate plasma free fatty acids (FFA) by 1.5–2 fold as previously described^6,8^, (3) oleate + the SIRT1 activator resveratrol (OLE + RSV) (RSV; 0.025 mg/kg.min, Sigma, St. Louis, MO, USA), or (4) RSV alone. Oleate was prepared in fatty acid free BSA as described previously^15^. Saline was used as a control as we have previously shown no difference from the BSA vehicle^9,15^ using the same protocol. The resveratrol was prepared as in previous studies in our laboratory^16^. Briefly, resveratrol was dissolved in 20% cyclodextrin (dissolved in saline; Sigma, St. Louis, MO, USA) and the pH was adjusted to 7.4. The solution was protected from light. The rate of resveratrol infusion was half of that used for 7 h duration in a previous study by our laboratory where RSV prevented fat-induced insulin resistance^16^. In that study, the plasma levels of RSV were close to those achieved by oral supplementation in humans^17^.

#### In vivo GSIS

To evaluate glucose-stimulated insulin secretion we used the hyperglycemic clamp method. This method allows assessment of glucose tolerance from the glucose infusion rate that is required to achieve and maintain the hyperglycemic target. It is a superior method to the more commonly used IPGTT because the hyperglycemic clamp involves maintaining a steady state of glucose elevation equal for all experimental groups. This steady state of hyperglycemia allows for calculation of an insulin sensitivity index and a disposition index (see Calculations), the latter being essential to the evaluation of β-cell function in vivo when there are changes in insulin sensitivity (mice vs. rats in the present study). Two-step hyperglycemic clamp studies were performed in overnight fasted, conscious rats at the end of the 48 h infusion period as previously described^15^. GSIS was evaluated from the plasma insulin and C-peptide response to the rise in plasma glucose. Glucose levels were ‘clamped’ at 13 mM (upper physiological glucose level for rats) for 120 min and then at 22 mM (maximum stimulatory levels) by a variable glucose infusion adjusted according to frequent (every 5–10 min) glycemic readings. Samples for insulin, C-peptide, and FFA were taken at regular intervals.

### Studies in mice

#### Surgery

An indwelling catheter was inserted into a jugular vein of mice for i.v. infusions as previously described^8,9^. After 3–4 days post-surgery recovery the WT and TG mice were randomized to the following 48 h i.v. infusions (1) SAL (WTSAL; TGSAL) or (2) OLE (WTOLE; TGOLE) to elevate plasma NEFA by 1.5–2 fold. Oleate was prepared as above and was administered at 0.4 μmol/min.

#### Beta cell function in vivo

One-step hyperglycemic clamps (22 mM), which are less invasive than the two-step clamps performed in rats, were performed as previously described^9^ in conscious mice fasted for 4 h. The glucose infusion was given through the jugular catheter. The tail vein was used for sampling. Samples for insulin, C-peptide, and FFA were taken at basal and during the last 20 min of the hyperglycemic clamp.

### Plasma assays

Plasma glucose in rats was measured with a Beckman 2 or Analox GM9 glucose analyzer. In mice, plasma glucose readings were obtained on a Hemocue glucose analyzer (HemoCue, Lake Forest, CA). Plasma FFA levels were measured by enzymatic colorimetric kit (Wako Industries, Neuss, Germany). Insulin and C-peptide were measured using rat/mouse-specific Radioimmunoassay kits (Linco Research Inc., MO, USA).

### Calculations

#### Insulin sensitivity index (M/I)

The insulin sensitivity index (M/I) was calculated during the last 30 min of each step of the hyperglycemic clamp in rats and during the last 30 min of the 22 mM hyperglycemic clamp in mice according to the following formula: M/I = Ginf/Insulin, where GINF is the rate of glucose infusion and Insulin is the plasma insulin concentration. This equation assumes that the change in glucose uptake and production induced by a change in plasma insulin is proportional to the ambient insulin concentration, which may not be a valid assumption at maximal insulin concentration^18^. Although we recognize this limitation, performing hyperinsulinemic clamps (the gold-standard method of assessment for insulin sensitivity) would have required another set of animals because it is too invasive to perform both hyperglycemic and hyperinsulinemic clamps in the same animal.

#### Disposition index (DI)

In vivo, there is a hyperbolic relationship between insulin sensitivity and insulin secretion^19^, which we have validated in rodents^20,21^. To account for changes in sensitivity, β-cell secretory function in vivo should be assessed by taking into account β-cell compensation for insulin resistance, which follows the hyperbola described above. The DI, i.e., the constant of the hyperbola, is the best established index of β-cell function in vivo^22^, and was calculated at each step of the hyperglycemic clamp according to the following formula: DI = M/I*C-peptide, where M/I is calculated as described above and C-peptide is the C-peptide concentration (index of absolute insulin secretion) during the last 30 min of each step of the hyperglycemic clamp in rats and during the last 30 min of the 22 mM hyperglycemic clamp in mice. The insulin secretion rate cannot be calculated in rodents as the kinetics of C-peptide (species specific and unavailable for injection) are unknown.

#### Insulin clearance

The steady-state C-peptide to insulin ratio during the clamp was used as an index of insulin clearance as C-peptide is co-secreted with insulin and cleared by the kidney proportionally to its concentration.

### Statistical analysis

Data are presented as means ± SEM. One-way non-parametric analysis of variance (ANOVA) for repeated measurements followed by Tukey’s test was used to compare differences between treatments. Criteria for non-parametric ANOVA (homogeneity of rank variances) were met. Sample size was estimated based on a power of 0.8 to detect a 50% difference in DI. Experiments were excluded if technical difficulties occurred prior to or during the first stage of the hyperglycemic clamp. There was no blinding. Calculations were performed using SAS (Cary, NC, USA).

## Results

### Studies in rats

#### FFA levels are increased after 48 h oleate infusion

Following the 48 h infusions, plasma FFA levels of both the OLE and OLE + RSV groups were significantly elevated (*p* < 0.01) compared to the SAL control and RSV alone groups (Table 1).

**Table 1:** Plasma FFA levels before and after the 48 h infusion period

Group	Time (h)	FFA (μM ± SEM)	Significance (vs. SAL)
SAL	0	634 ± 83	–
	48	660 ± 62	–
OLE	0	663 ± 101	NS
	48	1026 ± 97	*p* < 0.01
OLE + RSV	0	633 ± 39	NS
	48	995 ± 63	*p* < 0.01
RSV	0	615 ± 92	NS
	48	711 ± 56	NS

Rats were infused with: (1) saline (SAL, *n* = 6), (2) oleate alone (OLE, *n* = 7) at 1.4 µmol/min, (3) OLE + resveratrol at 0.025 mg/kg min (OLE + RSV, *n* = 6), and (4) RSV alone (RSV *n* = 8). Data are means ± SEM

#### Resveratrol partially prevents oleate-induced β-cell dysfunction during a two-step hyperglycemic clamp

**Fig. 1:**
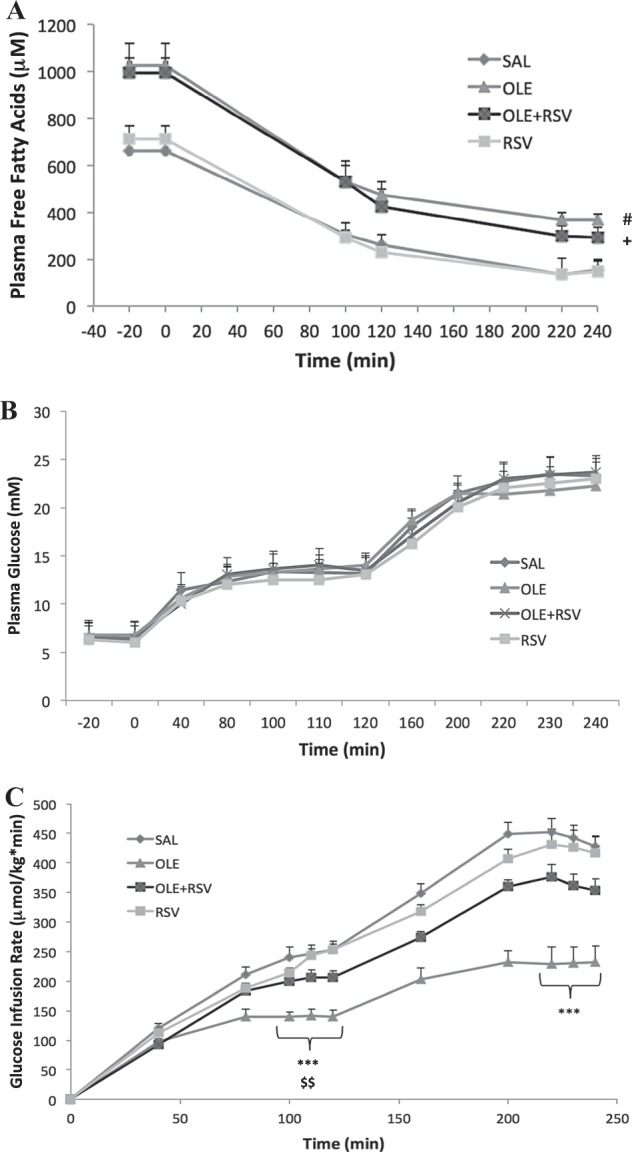
Resveratrol partially prevents oleate-induced decrease in glucose infusion rate during a hyperglycemic clamp. Plasma FFA levels (**a**), plasma glucose levels (**b**), and glucose infusion rate (Ginf) (**c**) during the two-step hyperglycemic clamp. Rats were infused with: (1) Saline (SAL, *n* = 8), (2) Oleate alone (OLE, *n* = 7) at 1.4 µmol/min, (3) OLE + Resveratrol at 0.025 mg/kg min (OLE + RSV, *n* = 6), and (4) RSV alone (RSV, *n* = 9). Data are means ± SEM. **a** ^#^*p* < 0.05 OLE vs. SAL and RSV throughout,^+^*p* < 0.05 OLE + RSV vs. SAL and RSV basal state and 2nd step of clamp. **c** ***p < 0.001 OLE vs. SAL, ^$$^*p* < 0.01 OLE vs. RSV

During the clamp period, plasma FFA levels in all groups decreased (Fig. 1a), as expected, due to hyperglycemia and hyperinsulinemia.

Basal plasma glucose levels prior to the start of the clamp did not significantly differ between groups. At time 0 min, glucose infusion was started and plasma glucose was gradually raised to 13 mM until 120 min and then to 22 mM until 240 min. There were no significant differences in plasma glucose levels between groups during the clamp (Fig. 1b).

The glucose infusion rate (Ginf), which reflects the amount of glucose infused to reach and maintain the hyperglycemic target, was significantly lower during both stages of the clamp in OLE-infused rats compared to both the SAL controls and RSV alone groups (Fig. 1c). The Ginf was lower in the OLE group whether normalized for body weight or expressed in absolute values because there was no difference in body weight among groups (Supplementary Table 1). The group infused with OLE + RSV had a partially restored Ginf compared to the SAL and OLE groups. There were no significant differences between SAL and RSV alone.

**Fig. 2:**
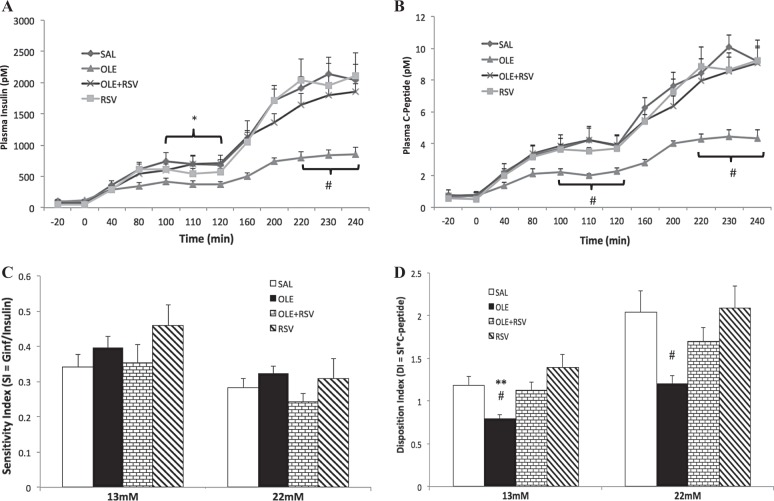
Resveratrol partially prevents oleate-induced decrease in β-cell function. Plasma insulin levels (**a**), plasma C-peptide levels (**b**), sensitivity index (**c**), and disposition index (**d**) during the two-step hyperglycemic clamp. Rats were infused with: (1) saline (SAL, *n* = 8), (2) oleate alone (OLE, *n* = 7) at 1.4 µmol/min, (3) OLE + resveratrol at 0.025 mg/kg min (OLE + RSV, *n* = 6), and (4) RSV alone (RSV, *n* = 9). Significance is indicated during the last 30 min of each clamp step. Data are means ± SEM. **a**–**d** ^#^*p* < 0.05 OLE vs. SAL and RSV, *p < 0.05 OLE vs. SAL, **p < 0.01 OLE vs. SAL

Basal plasma insulin and C-peptide levels were similar in all groups prior to the start of the clamp (−20 min; Fig. 2a, b). Upon starting the clamp, the rising glucose levels caused an increase in both plasma insulin and C-peptide levels as expected, reflecting increased secretion. Similar to the Ginf, both insulin and C-peptide levels were significantly lower in the OLE group during both steps of the clamp compared to the SAL control. There were no significant differences between the SAL, RSV alone, or OLE + RSV groups.

The sensitivity index (M/I index, see Methods) was not significantly different between any groups at either 13 or 22 mM (Fig. 2c). The DI, which more accurately reflects β-cell function in cases of insulin resistance (see Methods), was significantly lower in the OLE group compared to both SAL and RSV alone at both 13 and 22 mM (Fig. 2d). Since there were no changes in the sensitivity index, the decreased DI corresponds with the insulin or C-peptide levels and reflects a decrease in β-cell function in the OLE group compared to SAL. The OLE + RSV group had a restored DI at 13 mM and a partially restored DI at 22 mM that was not significantly different versus any other group. There were no significant differences between SAL and RSV alone.

Insulin clearance index (C-peptide/insulin, nM/pM) was not significantly different among groups (13 mM: SAL = 0.0055 ± 0.0005, OLE = 0.0059 ± 0.0005, OLE + RSV = 0.0064 ± 0.0006, RSV = 0.0069 ± 0.0008; 22 mM: SAL = 0.0048 ± 0.0006, OLE = 0.0055 ± 0.0003, OLE + RSV = 0.0049 ± 0.0003, RSV = 0.0055 ± 0.0009).

### Studies in mice

#### FFA levels are elevated following 48 h oleate infusion

**Fig. 3:**
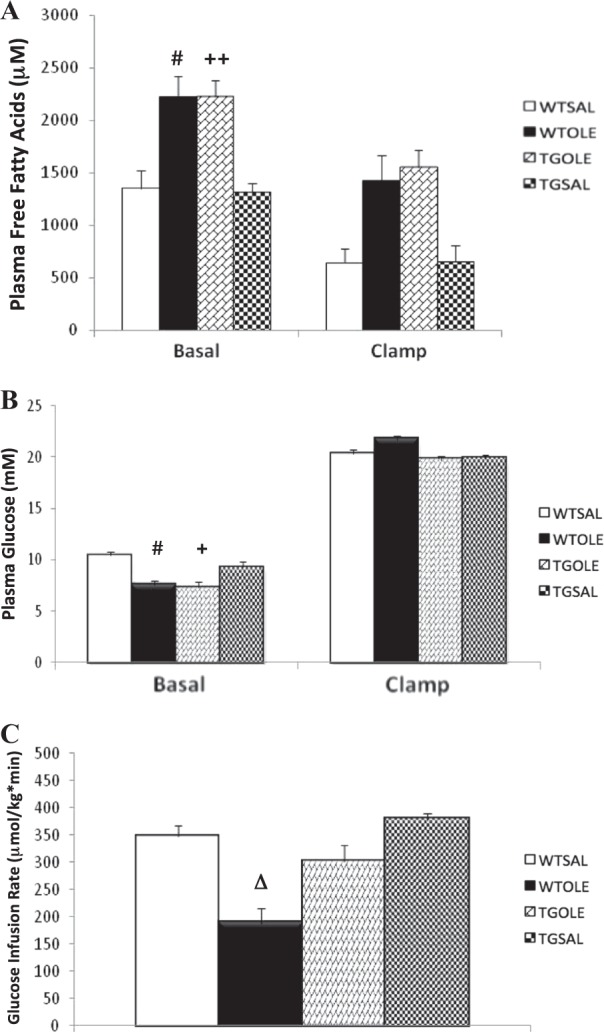
Oleate-induced decrease in glucose infusion rate in wildtype mice is partially prevented in BESTO mice. Plasma free fatty acid levels (**a**), plasma glucose levels (**b**), and glucose infusion rate (Ginf) (**c**) during the one-step hyperglycemic clamp. BESTO mice (TG) or wildtype (WT) littermates were treated for 48 h with either (1) saline (WTSAL, *n* = 8; TGSAL, *n* = 7) or (2) oleate at 3.9 μmol/min (WTOLE, *n* = 8; TGOLE, *n* = 8). Data are means ± SEM and refer to the last 30 min of the basal period and clamp. **a** ^#^*p* < 0.05 WTOLE vs. WTSAL and TGSAL, ^++^*p* < 0.01 TGOLE vs. TGSAL and WTSAL. **b** ^#^*p* < 0.05 WTOLE vs. WTSAL and TGSAL, ^+^*p* < 0.05 TGOLE vs. TGSAL and WTSAL. **c** Δ*p* < 0.05 vs. all

Following the 48 h infusions, plasma FFA levels of both the WTOLE and TGOLE groups were significantly elevated (*p* < 0.05) compared to the WTSAL and TGSAL groups (Table 2). FFA levels during the clamp were still elevated in the oleate-infused groups (Fig. 3a).

**Table 2:** Plasma FFA levels before and after the 48 h infusion period

Group	Time (h)	FFA (μM ± SEM)	Significance (vs. WTSAL/TGSAL)
WTSAL	0	1011 ± 160	–
	48	1354 ± 169	–
WTOLE	0	1281 ± 110	NS
	48	2222 ± 167	*p* < 0.05/*p* < 0.01
TGOLE	0	1299 ± 174	NS
	48	2229 ± 155	*p* < 0.01/*p* < 0.001
TGSAL	0	1161 ± 178	NS
	48	1319 ± 80	NS

BESTO mice (TG) or wildtype (WT) littermates were treated for 48 h with either (1) saline (WTSAL, *n* = 6; TGSAL, *n* = 7) or (2) oleate at 3.9 μmol/min (WTOLE, *n* = 8; TGOLE, *n* = 8). Data are means ± SEM

#### Oleate-induced β-cell dysfunction is partially prevented in BESTO mice

Basal plasma glucose levels prior to the start of the clamp were slightly lower in both oleate-infused groups compared to both saline control groups (Fig. 3b). There were no differences between the two saline groups or between the two oleate groups. At time 0 min, glucose infusion was started and plasma glucose was raised to 22 mM until 120 min. There were no significant differences in plasma glucose levels between groups during the clamp (Fig. 3b).

The glucose infusion rate (Ginf) was significantly lower in the WTOLE group compared to all other groups (Fig. 3c). Again, the Ginf was lower in the WTOLE group whether normalized for body weight or expressed in absolute values because there was no difference in body weight among groups (Supplementary Table 2). The TGOLE group was partially protected from the oleate-induced decrease in Ginf, which was not significantly different from either the WTSAL or TGSAL groups. There was no significant difference between the WTSAL and TGSAL groups.

**Fig. 4:**
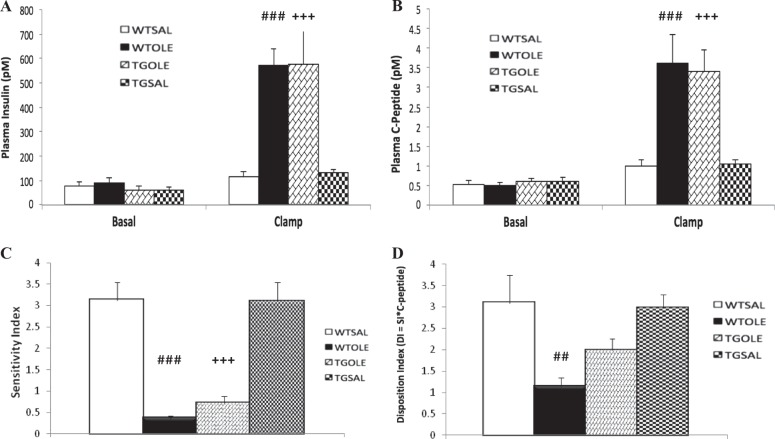
Oleate-induced decrease in β-cell function in wildtype mice is partially prevented in BESTO mice. Plasma insulin levels (**a**), plasma C-peptide levels (**b**), sensitivity index (**c**), and disposition index (**d**) during the one-step hyperglycemic clamp. BESTO mice (TG) or wildtype (WT) littermates were treated for 48 h with either (1) saline (WTSAL, *n* = 8; TGSAL, *n* = 7) or (2) oleate at 3.9 μmol/min (WTOLE, *n* = 8; TGOLE, *n* = 8). Data are means ± SEM and refer to the last 30 min of the basal period and clamp. **a**–**d** ^##^*p* < 0.01, ^###^*p* < 0.001 WTOLE vs. WTSAL and TGSAL, ^+++^*p* < 0.001 TGOLE vs. TGSAL and WTSAL

Basal plasma insulin and C-peptide levels were similar in all groups (Fig. 4a, b). During the clamp, both plasma insulin and C-peptide levels were elevated compared to basal levels across all groups. Compared to both SAL-treated groups, the OLE-treated groups had significantly higher insulin and C-peptide levels. This elevation is due to insulin resistance induced by oleate treatment in mice^9^. There were no significant differences between the two SAL-treated groups or the two OLE-treated groups in either insulin or C-peptide.

The sensitivity index (M/I index, see Methods for details) was significantly lower in both OLE-treated groups compared to the SAL-treated groups (Fig. 4c). The TGOLE group appeared to have slightly improved insulin sensitivity compared to the WTOLE group, however this difference was not significant. There was no significant difference in sensitivity index between the WTSAL and TGSAL groups. The disposition index (DI) was significantly lower in the WTOLE group compared to both the WTSAL and TGSAL groups (Fig. 4d). In this case, the DI more accurately reflects β-cell function compared to insulin/C-peptide levels since it takes into account the insulin resistance caused by the oleate treatment. The TGOLE group was partially protected from the oleate-induced β-cell dysfunction as evident from an intermediate DI compared to both saline groups. There was no significant difference between the TGOLE group and any other group. There was also no significant difference between the two saline groups.

The insulin clearance index (C-peptide/insulin, nM/pM) was not significantly different among groups (WTSAL = 0.0096 ± 0.002, WTOLE = 0.0061 ± 0.0008, TGOLE = 0.0060 ± 0.0008, TGSAL = 0.0080 ± 0.007).

## Discussion

Here, we have attempted to further understand the role of SIRT1 in pancreatic β-cells by determining whether enhancing its activity affects β-cell function in vivo in conditions of decreased β-cell function selectively mediated by fat. In our studies in rats we used the SIRT1 activator resveratrol to attenuate the fat-induced decrease in β-cell function as assessed through hyperglycemic clamp. We found that resveratrol partially prevented the oleate-induced decrease in β-cell function in rats that were co-infused with both oleate and resveratrol. To further implicate SIRT1, in our second study we determined whether mice that overexpressed SIRT1 specifically within the β-cell were also protected from fat-induced decreased β-cell function. Once again we discovered that these TG mice displayed partial prevention of the fat-induced decrease in β-cell function. Altogether, these two studies demonstrated that activation of SIRT1 can at least partially prevent lipid-induced decrease in β-cell function in vivo.

In interpreting the above results, it is important to consider differences between our intravenous (i.v.) fat model and the more conventional high fat diet (HFD) model. First, compared to dietary fat, our i.v. infusion model is specific for circulating fat, in that it avoids gastrointestinal neural and endocrine factors affecting insulin secretion, as well as adipose tissue expansion with related changes in adipokine/cytokine secretion. Being less chronic than HFD, our i.v. fat infusion model is also a selective model of decreased β-cell function as β-cell mass is not decreased^9,23^ and apoptosis is not affected^24^. Oleate is generally considered less toxic than palmitate for β-cells and in small amounts may confer health benefits in connection to the Mediterranean diet. However, part of the benefit of the Mediterranean diet can be attributed to antioxidants present in virgin olive oil. In addition, orally administered oleate can increase insulin secretion via stimulating incretin release^25^. We gave i.v. infusion of oleate to mimic lipolytic release of fatty acids as in obesity and Type 2 diabetes and there is evidence within the literature of decreased β-cell function with prolonged direct exposure of β-cells to oleate^26^. As lipolysis involves both oleate and palmitate, ideally both should be included, however there are not many studies with palmitate in vivo, primarily due to the challenges and toxicity of i.v. palmitate infusion^27^. One important consideration of all these models, including our own, is that decreased β-cell function may in part be an adaptive response to energy excess as less insulin is required to transport glucose into muscle due to excess fat. However, we have previously linked decreased β-cell cell function in our oleate infusion model with increased ROS and inflammation in islets^6–9,28^, which is suggestive of lipotoxicity. As published before^6,8,9,15,28^, we found that decreased β-cell cell function with oleate was not accompanied by fasting hyperglycemia, presumably indicating that initial reduction in β-cell cell function can only be detected by using β-cell challenge tests.

Although controversy remains as to whether resveratrol activates SIRT1 directly or indirectly^29–32^, there is no doubt resveratrol is an activator of SIRT1. More specific to β-cells, resveratrol has been shown in other studies to enhance GSIS^33^ and increase cellular ATP levels^33,34^. There is also evidence that SIRT1 mRNA and protein levels are decreased in conditions of energy excess, including Type 2 diabetes and obesity^35–37^. Having previously established a model of lipid-induced decrease in β-cell function^6^, here we attempted to explore whether resveratrol could prevent this decrease in rats. We confirmed that rats infused with oleate for 48 h had a significant decrease in β-cell function. In rats that were co-infused with both oleate and resveratrol we saw partial prevention of the oleate-induced decrease in β-cell function. In general, our findings are in line with a number of studies using HFD that have demonstrated improved glucose tolerance, improved levels of plasma glucose and insulin and enhanced GSIS with resveratrol treatment^38–40^. Most of these HFD studies assess only glucose tolerance without accounting for changes in insulin sensitivity and therefore the effect of resveratrol specifically on the β-cell remains unclear. However, a study by Wu et al. demonstrated decreased SIRT1 expression in vitro in palmitate-exposed rat islets that had reduced GSIS^13^. Adding resveratrol to the culture prevented this reduction in SIRT1 and β-cell function. They also showed that 24 h infusion of Intralipid in rats reduced SIRT1 mRNA and protein levels, but they did not assess prevention of this decrease using resveratrol.

The most obvious limitation in using resveratrol as a SIRT1 activator is its many nonspecific effects. For example resveratrol has antioxidant effects that may be independent of SIRT1^41,42^, although in our previous study i.v. resveratrol given for 7 h at double the infusion rate did not affect oxidative stress markers in muscle^16^. This is why we also investigated a genetic model of SIRT1 activation, the BESTO mouse, where our results are in accordance with those using resveratrol in rats and also in overall accordance with the results reported using HFD in BESTO mice^38–40^. However, our studies showed normal β-cell function in untreated BESTO mice, in contrast with the enhanced insulin secretion reported by Moynihan et al.^2^ The difference may relate to the fact that DI, being calculated from steady-state measures, represents mostly second phase insulin secretion, whereas the increase in insulin levels during the IPGTT used by Moynihan et al. may have reflected a significant increase in first-phase secretion.

The primary limitation of the BESTO mouse model is its high level of SIRT1 overexpression (up to 18×), which we confirmed in our colony (Supplementary Figure 1 and Supplementary Methods). This has been argued to exaggerate the beneficial results of SIRT1 overexpression that may not be apparent at lower levels of overexpression. However, subsequent whole body overexpression models displayed more moderate levels of SIRT1 overexpression but the results were generally concordant with the findings using the BESTO model^43–46^. Notably, despite the high level of SIRT1 overexpression, our studies revealed only a partial preservation of β-cell function in BESTO mice. One possible explanation for this finding is that SIRT1 activity is limited by NAD availability, which is likely lower during conditions of nutrient excess. Yoshino et al.^3^ addressed this notion by demonstrating that NAMPT-mediated NAD synthesis is compromised in HFD-fed glucose-intolerant mice. However, supplementation of NMN, a NAD precursor, can restore glucose tolerance.

The mechanism whereby SIRT1 increases β-cell function in the context of elevated fatty acids remains to be investigated. In addition to UCP2 suppression, improved mitochondrial function^5^, reduced ER stress as well as increased expression of GLUT2 and GLP1 receptor^4^ have been implicated in previous studies of SIRT1 effects in the absence of fatty acid elevation. In our recent studies we have shown that i.v. RSV prevents fat-induced insulin resistance via its anti-inflammatory effect on IKKβ^16^. Since we have also shown that IKKβ plays an important role in fat-induced decrease in β-cell function^28^, it is also possible that RSV and SIRT1 exert anti-inflammatory effects in β-cells.

In conclusion, our studies show that pharmacologic and genetic models of SIRT1 activation partially prevent the decrease in β-cell function specifically induced by circulating fat and therefore suggest SIRT1 as a therapeutic target to increase β-function in conditions where circulating fat is elevated, such as obesity and  Type 2 diabetes.

## Supplementary information

Supplemental Material - PDF File

## References

[1] Bordone L (2006). Sirt1 regulates insulin secretion by repressing UCP2 in pancreatic beta cells. PLoS Biol..

[2] Moynihan KA (2005). Increased dosage of mammalian Sir2 in pancreatic beta cells enhances glucose-stimulated insulin secretion in mice. Cell. Metab..

[3] Yoshino J, Mills KF, Yoon MJ, Imai S (2011). Nicotinamide mononucleotide, a key NAD(+) intermediate, treats the pathophysiology of diet- and age-induced diabetes in mice. Cell. Metab..

[4] Pinho AV (2015). Pancreas-specific Sirt1-deficiency in mice compromises beta-cell function without development of hyperglycemia. PLoS One.

[5] Luu L (2013). The loss of Sirt1 in mouse pancreatic beta cells impairs insulin secretion by disrupting glucose sensing. Diabetologia.

[6] Oprescu AI (2007). Free fatty acid-induced reduction in glucose-stimulated insulin secretion: evidence for a role of oxidative stress in vitro and in vivo. Diabetes.

[7] Giacca A, Xiao C, Oprescu AI, Carpentier AC, Lewis GF (2011). Lipid-induced pancreatic beta-cell dysfunction: focus on in vivo studies. Am. J. Physiol. Endocrinol. Metab..

[8] Koulajian K (2013). NADPH oxidase inhibition prevents beta cell dysfunction induced by prolonged elevation of oleate in rodents. Diabetologia.

[9] Koulajian K (2013). Overexpression of glutathione peroxidase 4 prevents beta-cell dysfunction induced by prolonged elevation of lipids in vivo. Am. J. Physiol. Endocrinol. Metab..

[10] Bai P (2011). PARP-1 inhibition increases mitochondrial metabolism through SIRT1 activation. Cell. Metab..

[11] Bai P (2011). PARP-2 regulates SIRT1 expression and whole-body energy expenditure. Cell. Metab..

[12] Xiong S, Salazar G, Patrushev N, Alexander RW (2011). FoxO1 mediates an autofeedback loop regulating SIRT1 expression. J. Biol. Chem..

[13] Wu L (2012). Activation of SIRT1 protects pancreatic beta-cells against palmitate-induced dysfunction. Biochim. Biophys. Acta.

[14] Santos L, Escande C, Denicola A (2016). Potential modulation of sirtuins by oxidative stress. Oxid. Med. Cell Longev..

[15] Mason TM (1999). Prolonged elevation of plasma free fatty acids desensitizes the insulin secretory response to glucose in vivo in rats. Diabetes.

[16] Pereira S (2015). Resveratrol prevents insulin resistance caused by short-term elevation of free fatty acids in vivo. Appl. Physiol. Nutr. Metab..

[17] Timmers S (2011). Calorie restriction-like effects of 30 days of resveratrol supplementation on energy metabolism and metabolic profile in obese humans. Cell. Metab..

[18] Natali A (2000). Dose–response characteristics of insulin action on glucose metabolism: a non-steady-state approach. Am. J. Physiol. Endocrinol. Metab..

[19] Kahn SE (1993). Quantification of the relationship between insulin sensitivity and beta-cell function in human subjects. Evidence for a hyperbolic function. Diabetes.

[20] Goh TT (2007). Lipid-induced beta-cell dysfunction in vivo in models of progressive beta-cell failure. Am. J. Physiol. Endocrinol. Metab..

[21] Tang C (2013). Susceptibility to fatty acid-induced beta-cell dysfunction is enhanced in prediabetic diabetes-prone biobreeding rats: a potential link between beta-cell lipotoxicity and islet inflammation. Endocrinology.

[22] Faerch K, Brons C, Alibegovic AC, Vaag A (2010). The disposition index: adjustment for peripheral vs. hepatic insulin sensitivity?. J. Physiol..

[23] Tang C (2012). Glucose-induced beta cell dysfunction in vivo in rats: link between oxidative stress and endoplasmic reticulum stress. Diabetologia.

[24] Poitout V (2010). Glucolipotoxicity of the pancreatic beta cell. Biochim. Biophys. Acta.

[25] Iakoubov R, Ahmed A, Lauffer LM, Bazinet RP, Brubaker PL (2011). Essential role for protein kinase Czeta in oleic acid-induced glucagon-like peptide-1 secretion in vivo in the rat. Endocrinology.

[26] Maris M (2011). Oleate-induced beta cell dysfunction and apoptosis: a proteomic approach to glucolipotoxicity by an unsaturated fatty acid. J. Proteome Res..

[27] Eguchi K (2012). Saturated fatty acid and TLR signaling link beta cell dysfunction and islet inflammation. Cell. Metab..

[28] Ivovic A (2017). IKKbeta inhibition prevents fat-induced beta cell dysfunction in vitro and in vivo in rodents. Diabetologia.

[29] Borra MT, Smith BC, Denu JM (2005). Mechanism of human SIRT1 activation by resveratrol. J. Biol. Chem..

[30] Kaeberlein M (2005). Substrate-specific activation of sirtuins by resveratrol. J. Biol. Chem..

[31] Pacholec M (2010). SRT1720, SRT2183, SRT1460, and resveratrol are not direct activators of SIRT1. J. Biol. Chem..

[32] Beher D (2009). Resveratrol is not a direct activator of SIRT1 enzyme activity. Chem. Biol. Drug Des..

[33] Vetterli L, Brun T, Giovannoni L, Bosco D, Maechler P (2011). Resveratrol potentiates glucose-stimulated insulin secretion in INS-1E beta-cells and human islets through a SIRT1-dependent mechanism. J. Biol. Chem..

[34] Price NL (2012). SIRT1 is required for AMPK activation and the beneficial effects of resveratrol on mitochondrial function. Cell. Metab..

[35] Gillum MP, Erion DM, Shulman GI (2011). Sirtuin-1 regulation of mammalian metabolism. Trends Mol. Med..

[36] Pedersen SB, Olholm J, Paulsen SK, Bennetzen MF, Richelsen B (2008). Low Sirt1 expression, which is upregulated by fasting, in human adipose tissue from obese women. Int. J. Obes..

[37] Yoneda M (2010). Decreased SIRT1 expression and LKB1 phosphorylation occur with long-term high-fat diet feeding, in addition to AMPK phosphorylation impairment in the early phase. Obes. Res Clin. Pract..

[38] Lagouge M (2006). Resveratrol improves mitochondrial function and protects against metabolic disease by activating SIRT1 and PGC-1alpha. Cell.

[39] Baur JA (2006). Resveratrol improves health and survival of mice on a high-calorie diet. Nature.

[40] Zhang J (2012). The protective effect of resveratrol on islet insulin secretion and morphology in mice on a high-fat diet. Diabetes Res. Clin. Pract..

[41] de la Lastra CA, Villegas I (2007). Resveratrol as an antioxidant and pro-oxidant agent: mechanisms and clinical implications. Biochem. Soc. Trans..

[42] Mahal HS, Mukherjee T (2006). Scavenging of reactive oxygen radicals by resveratrol: antioxidant effect. Res. Chem. Intermed..

[43] Banks AS (2008). SirT1 gain of function increases energy efficiency and prevents diabetes in mice. Cell. Metab..

[44] Bordone L (2007). SIRT1 transgenic mice show phenotypes resembling calorie restriction. Aging Cell.

[45] Herranz D (2010). Sirt1 improves healthy ageing and protects from metabolic syndrome-associated cancer. Nat. Commun..

[46] Pfluger PT, Herranz D, Velasco-Miguel S, Serrano M, Tschop MH (2008). Sirt1 protects against high-fat diet-induced metabolic damage. Proc. Natl Acad. Sci. USA.

