# Supplementary material for: Pharmacologic or genetic activation of SIRT1 attenuates the fat-induced decrease in beta-cell function in vivo
Source: Nutr Diabetes. 2019 Mar 19;9:11. doi: 10.1038/s41387-019-0075-z (PMC6424971; doi:10.1038/s41387-019-0075-z)
Supplement: Supplementary file 1 — Supplemental Material - PDF File [file 41387_2019_75_MOESM1_ESM.pdf]

Supplementary Tables and Figures

Supplementary Table 1.

Group	Body Weight (g ± SEM)	Significance (vs. SAL)
SAL	273.13 ± 7.28	-
OLE	275.43 ± 7.09	NS
OLE+RSV	283.00 ± 7.17	NS
RSV	269.67 ± 5.25	NS

Supplementary Table 1. Body weight by experimental group in rats. Female Wistar rats aged between 10-12 weeks were studied. Rats were infused with: 1) Saline (SAL, n=6), 2) Oleate alone (OLE, n=7) at 1.4 µmol/min, 3) OLE + Resveratrol at 0.025 mg/kg.min (OLE+RSV, n=6) and 4) RSV alone (RSV n=8). Data are means ± SEM.

Supplementary Table 2.

Group	Body Weight (g ± SEM)	Significance (vs. SAL)
WTSAL	25.45 ± 1.69	-
WTOLE	27.63 ± 0.52	NS
TGOLE	27.71 ± 0.99	NS
TGSAL	28.21 ± 0.68	NS

Supplementary Table 2. Body weight by experimental group in mice. BESTO mice (TG) and wildtype (WT) littermates aged 12-16 weeks were studied. (TG) or (WT) mice were treated for 48 h with either 1) saline (WTSAL, n=6; TGSAL, n=7) or 2) oleate at 3.9 µmol/min (WTOLE, n=8; TGOLE, n=8). Data are means ± SEM.

Supplementary Figure 1.

A

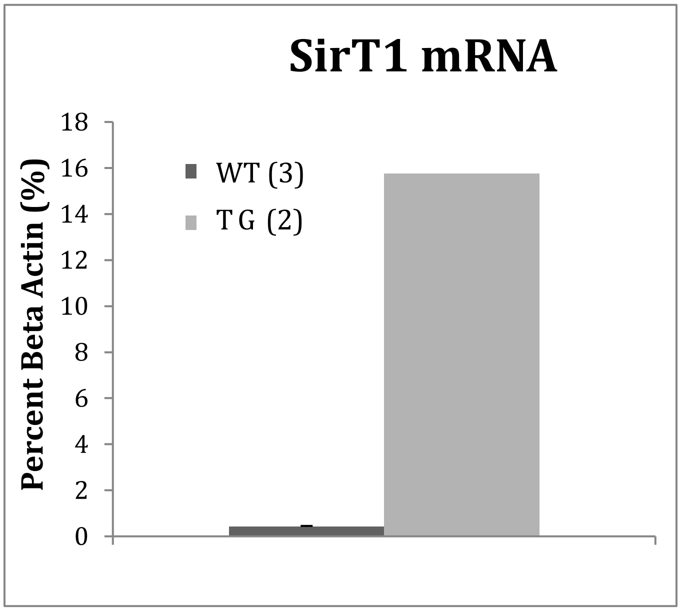

B

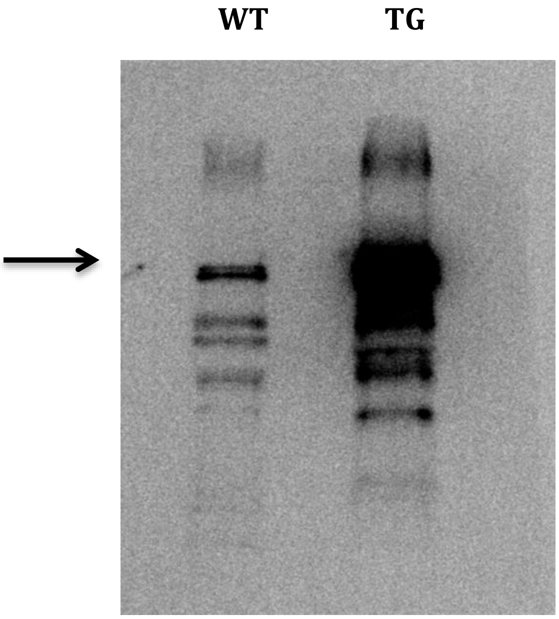

Supplementary Figure 1. Islet SIRT1 expression levels in TG and WT mice. (A) SIRT1 mRNA. (B) SIRT1 protein. SIRT1 mRNA was assessed through qPCR and TG mice had approximately 16 fold higher levels compared to WT mice. Protein levels were assessed through Western blot and were approximately 15 fold higher in TG mice compared to WT littermates. A representative blot is shown in (B), the arrow indicates the SIRT1 band.

Supplementary Methods

Mouse Islet Isolation. Pancreatic islets were isolated from male TG and WT mice as previously described (1). Briefly, mice were anesthetized with ketamine:xylazine:acepromazine (87:1.7:0.4 $mg.ml^{-1}$, $1 \mu l.g^{-1}$ of body weight). The visceral contents were exposed and mice were exsanguinated through an incision in the abdominal aorta. The pancreatic duct was perfused with 3 ml of collagenase V (0.8 mg/ml; Sigma, St. Louis, MO, USA) in RPMI-1640 containing 2.8mmol/l glucose, 10 mmol/l Hepes, 1% Penicillin. The pancreas was then excised and digested for 20 minutes at 37 °C. Islets were hand-picked from acinar tissue debris, and transferred into Krebs Ringer buffer containing 10mmol/l HEPES (KRBH) and 2.8 mmol/l glucose.

Western Blotting. Pancreatic islets were isolated as described above. Approximately 100-150 isolated mouse islets were hand-picked and washed with PBS prior to lysis directly in RIPA buffer containing protease inhibitor cocktail (Roche Diagnostics, Laval, QC, CAN). The cell lysates were then spun at 12,000rpm and the supernatant was loaded onto 4-15% SDS-PAGE gradient gel (Biorad, Hercules, CA, USA) and transferred onto PVDF membrane using the Turbo Blotter Transfer System (Biorad, Hercules, CA, USA). The membrane was probed with anti-SIRT1 antibody (Thermo Scientific) at a 1:500 dilution and imaged using a Kodak imager 4000pro and Carestream Imaging Software (Carestream, Rochester, NY, USA). Images were then quantified using Image J software.

RT-PCR and real-time PCR. Pancreatic islets were isolated as described above. Approximately 50-100 isolated mouse islets were hand-picked and washed with PBS. The total RNA was extracted from isolated mouse islets using an RNeasy Kit (Qiagen, Germany) and converted to cDNA using SuperScript II reverse transcriptase. The real-time PCR was performed as previously described (2). RT-PCR was performed using platinum Taq DNA polymerase on the Dual Block DNA Engine Thermal Cycler (MJ Research, Inc., MA, USA). The software used for real-time PCR primers is Primer Express (Applied Biosystems, CA, USA) and for RT-PCR primers is Primer3.

Supplementary References

1. Diao,J, Allister,EM, Koshkin,V, Lee,SC, Bhattacharjee,A, Tang,C, et al. UCP2 is highly expressed in pancreatic alpha-cells and influences secretion and survival. Proc Natl Acad Sci. 2008; 105:12057-12062.

2. Dai,FF, Zhang,Y, Kang,Y, Wang,Q, Gaisano,HY, Braunewell,KH, et al. The neuronal Ca2+ sensor protein visinin-like protein-1 is expressed in pancreatic islets and regulates insulin secretion. J Biol Chem. 2006; 281:21942-21953.
